# Occupational exposure to magnetic fields and breast cancer among Canadian men

**DOI:** 10.1002/cam4.581

**Published:** 2016-01-21

**Authors:** Anne Grundy, Shelley A. Harris, Paul A. Demers, Kenneth C. Johnson, David A. Agnew, Canadian Cancer Registries Epidemiology Research Group, Paul J. Villeneuve

**Affiliations:** ^1^ Department of Cancer Epidemiology and Prevention ResearchAlberta Health Services – Cancer Control AlbertaCalgaryAlbertaCanada; ^2^ Prevention and Cancer ControlCancer Care OntarioTorontoOntarioCanada; ^3^ Occupational Cancer Research CenterCancer Care OntarioTorontoOntarioCanada; ^4^ Division of Occupational and Environmental HealthDalla Lana School of Public HealthUniversity of TorontoTorontoOntarioCanada; ^5^ Division of EpidemiologyDalla Lana School of Public HealthUniversity of TorontoTorontoOntarioCanada; ^6^ Department of Epidemiology and Community MedicineUniversity of OttawaOttawaOntarioCanada; ^7^ University of Ontario Institute of TechnologyOshawaOntarioCanada; ^8^ Department of Health SciencesCarleton UniversityOttawaOntarioCanada

**Keywords:** Case–control study, magnetic fields, male breast cancer, occupational exposure

## Abstract

Occupational magnetic field (MF) exposure has been suggested as a risk factor for breast cancer in both men and women. Due to the rarity of this disease in men, most epidemiologic studies investigating this relationship have been limited by small sample sizes. Herein, associations of several measures of occupational MF exposure with breast cancer in men were investigated using data from the population‐based case–control component of the Canadian National Enhanced Cancer Surveillance System. Lifetime job histories were provided by 115 cases and 570 controls. Average MF exposure of individual jobs was classified into three categories (<0.3, 0.3 to <0.6, or ≥0.6 *μ*T) through expert blinded review of participant's lifetime occupational histories. The impact of highest average and cumulative MF exposure, as well as exposure duration and specific exposure‐time windows, on cancer risk was examined using logistic regression. The proportion of cases (25%) with a highest average exposure of ≥0.3 *μ*T was higher than among controls (22%). We found an elevated risk of breast cancer in men who were exposed to ≥0.6 *μ*T (odds ratio [OR] = 1.80, 95% CI = 0.82–3.95) when compared to those with exposures <0.3 *μ*T. Those exposed to occupational MF fields for at least 30 years had a nearly threefold increase in risk of breast cancer (OR = 2.77, 95% CI = 0.98–7.82) when compared to those with background levels of exposure. Findings for the other time‐related MF variables were inconsistent. Our analysis, in one of the largest case–control studies of breast cancer in men conducted to date, provides limited support for the hypothesis that exposure to MF increases the risk breast cancer in men.

## Introduction

While breast cancer is one of the most common cancers among women, it rarely occurs in men. In Canada, breast cancer accounted for less than 1% of newly diagnosed cancer cases in men in 2014 1. However, multiple reports have suggested that the incidence of breast cancer in men has been slowly increasing over the past several decades 2, 3, 4, 5. Aside from reproductive history, men and women share many of the same risk factors for breast cancer including age, family history, obesity, and ionizing radiation exposure 6, 7. In addition, disorders associated with estrogen/androgen imbalances such as Klinefelter syndrome, appear to increase the risk of breast cancer in men 6, 7, however, overall accounts for few cases.

There are important advantages to studying the relationship between occupational and environmental exposures and breast cancer in men as they have far fewer reproductive risk factors than women. For example, the risk of breast cancer in women is influenced by age at menarche, age at menopause, and several other reproductive characteristics (parity, age at first birth, oral contraceptive use), and hormone replacement therapy 8. Occupational exposure to magnetic fields (MF) is a suspected risk factor for breast cancer in men and the International Agency for Research on Cancer has classified extremely low‐frequency MFs as a possible carcinogen based largely on epidemiological studies of leukemia 9. One U.S. case–control study (227 cases, 300 controls) observed an increased risk of breast cancer among men exposed to MF in their job (odds ratio [OR] = 1.8, 95% CI: 1.0–3.7) relative to those with only background levels of exposure; the strongest relationship was found among those employed in electric trades and related occupations (OR = 6.0, 95% CI: 1.7–2.1) 10. However, results from several other case–control studies of men with smaller numbers of cancer cases found no associations between MF exposure and breast cancer 11, 12, 13, 14, 15, with similar results for studies estimating exposure based on occupational title 16, 17. Results from cohort studies of men are also mixed, with some detecting an elevated risk of breast cancer 18, 19, 20, 21, while others have had nonsignificant or null findings 22, 23, 24, 25, 26, 27. An important limitation of this work was that individual studies have had small numbers of exposed breast cancer cases, typically less than 10 11, 12, 14, 15, and thus were severely underpowered to detect possible associations. That a lack of statistical power may have masked true associations between MF exposure and male breast cancer is supported by findings from two meta‐analyses that found an overall increased risk when data across multiple studies were pooled 28, 29. Furthermore, many studies have only examined ever/never exposure to occupational MFs 14, 15, 16, 19, 22, 23, 24, 27, and few studies have evaluated the impact of specific exposure‐time windows 10, 17. MFs have been hypothesized to influence cancer risk through promoting effects on the growth of tumors 30, thus, the ability to investigate the impact of exposure characteristics such as duration may be important in assessing the relationship between MFs and breast cancer risk in men.

A greater number of studies have examined associations of MF exposure with breast cancer in women. According to literature summaries found in the IARC Monograph 9, a World Health Organization report 31, and two meta‐analyses 28, 32, these studies have tended to focus on residential MFs and electric blanket use as sources of exposure. Far fewer studies cited in these reports have investigated occupational exposure to MFs and breast cancer in women. While these studies of residential exposures do not provide support for the hypothesis that MFs increase the risk of breast cancer 28, 32, 33, 34, 35, it is important to note that these exposures are lower than in many workplace settings 34. In the occupational studies that have been conducted in women, while some work has detected or suggested an increased risk of female breast cancer 34, 35, 36, 37, 38, 39, 40, 41, other studies found no association 42, 43, 44, 45, 46, 47 and these null findings were supported by the results of a 2010 meta‐analysis 32. Studies of occupational MFs and breast cancer in women are limited by the relatively small number of women exposed to MFs in an occupational setting. Moreover, it is not straightforward in women to disentangle the effects of MF exposures from those of other established risk factors. Thus, since men may have been more likely to have held jobs with exposures to MFs and have far fewer established risk factors, studies of occupational MF exposure and breast cancer can provide important insights. The primary objective of this study was to evaluate associations between occupational exposures to MFs and breast cancer in men. An important component to addressing this objective was the evaluation of how different exposure latency periods modified these associations.

## Methods

This study population consisted of participants from the Canadian National Enhanced Cancer Surveillance System (NECSS). The NECSS was a population‐based case–control study conducted among men and women aged 20–74 from eight of 10 Canadian provinces (Alberta, British Columbia, Manitoba, Newfoundland and Labrador, Nova Scotia, Ontario, Prince Edward Island, and Saskatchewan). A detailed description of the design of the NECSS has been published previously 48. Briefly, a total of 20,730 Canadians with one of the 19 types of cancer and 5073 population‐based controls were recruited between 1994 and 1998. All NECSS participants completed a self‐administered questionnaire where they provided information concerning demographic characteristics such as age, ethnicity, marital status, education, and household income; lifestyle characteristics including smoking behaviors, diet, physical activity, and alcohol consumption; occupational and residential histories; and history of exposure to potential occupational and environmental carcinogens including exposure to “radiation sources” at home or at work. In the occupational history specifically, participants were asked to list all jobs they had ever had for at least 12 months (including seasonal and part‐time work) and for each job to specify the start and stop dates, the type of industry or business, and their main job duties.

A total of 115 incident participating cases of breast cancer in men were obtained from provincial cancer registries as described previously 48. As described previously, the response rate among cases was ~68% 48. The NECSS recruited controls from the general population 48. In five provinces (Prince Edward Island, Nova Scotia, Manitoba, Saskatchewan, and British Columbia) controls were identified from provincial health insurance plans, which covered up to 95% of provincial residents. In the other three provinces, recruitment occurred using random‐digit dialing (Newfoundland and Labrador and Alberta) and property assessment data (Ontario). Controls were frequency matched to the overall case group (including all cancer sites) by age and sex, with the aim of having one control per case within each sex and 5‐year age group for every cancer type in each province. Controls in our study were a subset of the full NECSS control group that had previously been used in a study of MF exposure and brain cancer 30, and whose lifetime occupational exposure history for MFs had been characterized. These controls, originally selected for the brain cancer analysis and used again here, were randomly selected from the full NECSS control group, age‐matched within 1 year of the brain cancer cases 30. As described previously, the response rate for the control population was ~65% 30.

Occupational exposure to MFs was assessed by expert review (D. A. A.), blinded to case–control status, as described previously 30. Specifically, this review considered information on potential exposures from an open literature review, personal communications, data collected in the Tri‐Utility Occupational Study 13, and measurements taken in various work environments in Ontario. When possible, data concerning the type of work and industry were considered for individual occupations. To maintain case–control blinding during coding of the breast cancer cases for this analysis, a subset of controls included in the brain cancer study 30 was included, however the original MF exposure data from the brain cancer study was used for all controls. MF exposure coding for the breast cancer cases was conducted at the same time as coding for the brain cancer analysis and identical exposure coding procedures were used. A list of all occupations for cases and controls was compiled and an exposure value based on time‐weighted average magnetic flux density for full‐time workers was given to each occupation. Average MF exposure for each occupation was grouped into three categories: <0.3, 0.3 to <0.6, and ≥0.6 *μ*T. As described previously 30, the 0.3 *μ*T cut‐point for MF exposure was based on the distribution of residential exposures from a previous Canadian study of residential MF exposures and childhood leukemia 49, where 0.3 *μ*T was estimated as the 82nd percentile for adult exposures in the same homes 50 and was chosen to provide reasonable assurance that occupational exposures were above the background levels expected from residential sources. Furthermore, as described by Villeneuve et al. 30, the upper cut‐point of 0.6 *μ*T was chosen to be double the 0.3 *μ*T, where highly exposed jobs included, among others, sheet metal workers, electricians, and electric utility workers (Table 1). MF exposures could not be classified for a total of 10 (0.5%) of jobs among controls and 100 (18.2%) jobs among cases. For the main analyses, individual jobs that were not classified were coded as unexposed for both case and control groups to maximize available sample size for breast cancer cases, and the influence of this assumption was tested via sensitivity analysis.

**Table 1:** Examples of jobs with high magnetic field (MF) exposure

High (≥0.6 *μ*T) MF exposure jobs
Sheet metal workers
Telephone cable splicer
Projectionists (motion pictures)
Welders
Electricians
Electronic assemblers
Electric utility workers

### Statistical analysis

Descriptive characteristics of cases and controls for known or suspected risk factors were expressed using means and standard deviations for continuous variables, and percentages for categorical variables. Differences between cases and controls were assessed using *t*‐tests and chi‐square tests for continuous and categorical variables, respectively. For all analyses, *P*s < 0.05 were considered statistically significant.

Several MF exposure metrics were constructed for the analyses. The highest average exposure to occupational MFs was classified as none (jobs with exposure less than 0.3 *μ*T), 0.3 to <0.6, and ≥0.6 *μ*T 30. Cumulative MF exposure was calculated using the same method as the brain cancer study 30, by using a MF index (MF index) which accounted for intensity of MF exposure (*E*), duration of employment (*D*), and whether employment was full or part time (*F*) for each job, calculated using the formula:

$$\text{MF index}=\sum_{i=1}^{i=j}E_{i}\times D_{i}\times F_{i}$$

where *E*_*i*_ = 0 for jobs <0.3 *μ*T, 1 for jobs 0.3 to <0.6 *μ*T, 2 for jobs ≥0.6* μ*T; *D*_*i*_, duration of employment (years); *F*_*i*_, 1 for full time, 0.5 for part time or seasonal employment; *j*, total number of jobs held.

To evaluate the impact of assigning weight to the intensity of MF exposure in a linear fashion in the MF index, a sensitivity analysis was also conducted assigning a weight of 4 to jobs in the ≥0.6 *μ*T exposure group, similar to the method used by Koemen et al. 51. Duration of exposure (number of years) in jobs above the 0.3 *μ*T average exposure threshold, the influence of time since most recent (last) exposure, time since first exposure, and age at first exposure to MFs were also considered in separate models.

Associations between MF exposure and breast cancer were evaluated using unconditional logistic regression. To evaluate the potential for confounding of these relationships by other breast cancer risk factors, three models were created: an unadjusted model, a model adjusted for age only, and a multivariate model that included risk factors identified a priori. Variables included in the full multivariate model were age (continuous), body mass index (BMI, kg/m^2^), and leisure‐time physical activity. Leisure‐time physical activity levels were evaluated based on the self‐reported number of hours of strenuous activity per month and split into tertiles on the basis of the distribution among controls. BMI was characterized using a categorical variable (<25 = normal, 25–29 = overweight, ≥30 = obese) 52. A quadratic age term was used to assess linearity, however it was not retained in the final model as it was not statistically significant and did not impact our OR estimates. For the same reason, socioeconomic characteristics (e.g., education, income, and marital status) were not included in the final model. Finally, a sensitivity analysis excluding all jobs whose MF exposure could not be classified was also conducted to evaluate the impact of including these in the unexposed group. All analyses were conducted using SAS, Version 9.2 (SAS Institute, Cary, NC).

## Results

The characteristics of the study's 115 cases and 570 controls are presented in Table 2. Briefly, men diagnosed with breast cancer were generally older, less educated, had a slightly higher BMI, and lower levels of strenuous physical activity when compared to controls. No differences were observed between cases and controls in the proportion of individuals who reported having worked with radiation sources either at home or at work.

**Table 2:** Descriptive characteristics of the incident breast cancer cases and controls, National Enhanced Surveillance System

Characteristic	Case (*N* = 115)	Control (*N* = 570)	Odds ratio (95% CI)	*P*‐value^a^
	Mean (SD)/*N* (%)	Mean (SD)/*N* (%)		
Age	58.5 (12.9)	50.6 (13.8)	1.05 (1.03–1.07)	<0.0001
Ethnicity				
European/Caucasian	107 (93.0%)	509 (90.4%)	1.00 (ref)	
Other	8 (7.0%)	54 (9.6%)	0.71 (0.33–1.52)	0.37
Education (total no. years)	11.8 (3.9)	12.8 (3.6)	0.93 (0.88–0.99)	0.01
Household income				
<$30,000	39 (33.9%)	123 (21.5%)	1.67 (0.99–2.82)	0.005
$30,000–$49,999	30 (26.1%)	147 (25.8%)	1.00 (ref)	0.95
$50,000–$99,999	21 (18.3%)	146 (25.6%)	0.76 (0.42–1.38)	0.09
≥$100,000	6 (5.2%)	32 (5.6%)	0.99 (0.38–2.56)	0.87
Prefer not to answer	17 (14.8%)	100 (17.5%)	0.90 (0.47–1.70)	0.47
Missing	2 (1.7%)	22 (3.9%)		
Marital status				
Married/Common law	87 (75.7%)	438 (76.8%)	1.00 (ref)	0.73
Divorced	14 (12.2%)	43 (7.5%)	1.65 (0.87–3.15)	0.10
Widowed	5 (4.4%)	10 (1.8%)	2.54 (0.85–7.62)	0.08
Single	9 (7.8%)	73 (12.8%)	0.63 (0.30–1.30)	0.13
Other	0	2 (0.4%)		
Body mass index (BMI)	27.4 (5.1)	26.1 (4.0)	1.07 (1.02–1.11)	0.02
BMI categories^b^				
Normal (<25)	35 (30.7%)	220 (38.9%)	1.00 (ref)	0.07
Overweight (25–29)	57 (50.0%)	262 (46.4%)	1.36 (0.86–2.14)	
Obese (≥30)	22 (19.3%)	83 (14.7%)	1.66 (0.92–2.98)	
Physical activity (no. hours per month strenuous activity)				
Mean (non‐zero values)	9.59 (12.6)	12.15 (15.4)		0.11
Tertiles				
None	80 (69.6%)	343 (60.2%)	1.00 (ref)	0.06
0 to <2.19	14 (12.2%)	75 (13.2%)	0.80 (0.43–1.49)	0.77
2.19 to <12.69	12 (10.4%)	74 (13.0%)	0.70 (0.36–1.34)	0.45
≥12.69	9 (7.8%)	78 (13.7%)	0.50 (0.24–1.03)	0.09
Worked with radiation sources at home or work^a^	7 (6.1%)	40 (7.1%)	0.85 (0.37–1.95)	0.71

a *P*‐values calculated based on chi‐square tests for categorical variables and *t*‐tests for continuous variables.

b Adjusting these comparisons for age did not alter differences between cases and controls.

Relationships between occupational MF exposure metrics and breast cancer are presented in Table 3. No clear association was observed between the highest average MF exposure and the risk of breast cancer. While ORs were elevated in the highest exposure category (≥0.6 *μ*T) across all models (age‐adjusted and fully adjusted), all confidence intervals included unity, and no trend across increasing levels of MF intensity was evident.

**Table 3:** ORs for occupational exposure to MFs and breast cancer in men, NECSS

MF exposure variable	Cases, *N* (%)^a^	Controls, *N* (%)^a^	Age‐adjusted OR (95% CI)	Multivariate OR (95% CI)^b^
Highest average exposure				
None	86 (75)	446 (78)	1.0 (ref)	1.0 (ref)
0.3 to <0.6 *μ*T	19 (17)	94 (17)	0.91 (0.52–1.60)	0.90 (0.51–1.59)
≥0.6 *μ*T	10 (9)	29 (5)	1.83 (0.84–4.01)	1.80 (0.82–3.95)
			*P*‐trend = 0.33	*P*‐trend = 0.36
Cumulative MF exposure^c^				
None	75 (65)	450 (79)	1.0 (ref)	1.0 (ref)
0 to <0.8	11 (10)	40 (7)	1.78 (0.85–3.72)	1.80 (0.85–3.83)
≥8.0	13 (11)	79 (14)	0.86 (0.45–1.65)	0.85 (0.44–1.64)
Missing	16 (14)	1 (0.2)	*P*‐trend = 0.98	*P*‐trend = 0.95
Cumulative MF exposure (with 5‐year lag)^d^				
None	76 (66)	454 (80)	1.0 (ref)	1.0 (ref)
0 to <0.8	10 (7)	42 (7)	1.52 (0.71–3.23)	1.62 (0.75–3.49)
≥8.0	13 (11)	73 (13)	0.89 (0.46–1.71)	0.90 (0.47–1.75)
Missing	16 (14)	1 (0.2)	*P*‐trend = 0.96	*P*‐trend = 0.97
Time since last exposure				
Never exposed	87 (76)	447 (78)	1.0 (ref)	1.0 (ref)
<10 years	13 (11)	63 (11)	1.28 (0.66–2.47)	1.25 (0.64–2.45)
10–19 years	7 (6)	28 (5)	1.03 (0.43–2.50)	1.04 (0.43–2.53)
20–29 years	3 (3)	14 (2)	1.26 (0.35–4.59)	1.44 (0.40–5.24)
≥30 years	5 (4)	18 (3)	0.81 (0.29–2.30)	0.76 (0.27–2.16)
			*P*‐trend = 0.99	*P*‐trend = 0.96
Time since last exposure				
Never exposed	87 (76)	447 (78)	1.0 (ref)	1.0 (ref)
<5 years	10 (9)	49 (9)	1.35 (0.63–2.88)	1.33 (0.62–2.88)
5–9 years	4 (4)	24 (4)	0.92 (0.30–2.85)	0.90 (0.29–2.81)
≥10 years	20 (17)	81 (14)	1.02 (0.58–1.79)	1.02 (0.58–1.80)
			*P*‐trend = 0.67	*P*‐trend = 0.70
Time since first exposure				
Never exposed	87 (77)	447 (78)	1.0 (ref)	1.0 (ref)
<10 years	5 (4)	20 (4)	2.31 (0.80–6.66)	2.45 (0.83–7.26)
10–19 years	4 (4)	26 (5)	1.21 (0.40–3.70)	1.19 (0.39–3.61)
20–29 years	8 (7)	40 (7)	1.02 (0.46–2.29)	1.06 (0.47–2.38)
≥30 years	11 (10)	37 (7)	0.88 (0.42–1.84)	0.84 (0.40–1.77)
			*P*‐trend = 0.94	*P*‐trend = 0.87
Age at first exposure				
Under 20	13 (11)	45 (8)	1.39 (0.71–2.74)	1.36 (0.68–2.69)
20–34	9 (8)	65 (11)	0.72 (0.34–1.52)	0.71 (0.34–1.51)
≥35	6 (5)	13 (2)	1.84 (0.67–5.08)	1.96 (0.70–5.45)
			*P*‐trend = 0.72	*P*‐trend = 0.71
Duration of exposure^e^				
Never exposed	89 (77)	480 (84)	1.0 (ref)	1.0 (ref)
<15 years	14 (12)	60 (11)	1.29 (0.68–2.44)	1.30 (0.68–2.48)
15–29 years	5 (4)	21 (3)	1.17 (0.42–3.23)	1.20 (0.43–3.33)
≥30 years	7 (6)	9 (1)	2.68 (0.96–7.55)	2.77 (0.98–7.82)
			*P*‐trend = 0.11	*P*‐trend = 0.06

OR, odds ratio; MF, magnetic field; BMI, body mass index; NECSS, National Enhanced Cancer Surveillance System.

a Totals may not add to *n* = 115 (cases) or *n* = 570 (controls) due to missing data.

b Adjusted for age, BMI, and physical activity.

c Calculated as intensity of exposure × duration × full‐time status as in NECSS brain cancer analysis (Villeneuve et al. 30).

d All exposure in 5 years prior to study interview excluded.

e Part‐time and seasonal jobs weighted as half‐time of full‐time jobs.

When time‐related exposure variables were considered, results were more mixed. No clear associations between cumulative MF exposure (measured by MF index) and breast cancer in men were observed, as all confidence intervals included 1.0 (Table 3). In a pattern opposite to that observed with highest average MF exposure, ORs were higher in the moderate (0 to <8.0 MF index) cumulative exposure category than in the highest (≥8.0 MF index) cumulative exposure group. Results were similar for the sensitivity analysis using a MF index with a weight of 4 given to jobs in the highest exposure group. While ORs in highest cumulative exposure category were now marginally greater than those in the moderate cumulative exposure category (Table S1), all confidence intervals still included 1.0 and there remained no clear association between cumulative MF exposure and breast cancer in men. Furthermore, when the 5 years of exposure data prior to the study interview were excluded to account for a potential latency period, ORs were similar to those in the main cumulative exposure analysis.

None of the time since last exposure, time since first exposure, or age at first exposure to occupational MFs was associated with male breast cancer (Table 3). As a sensitivity analysis, time since last exposure was also characterized as <5, 5–9, and ≥10 years prior to diagnosis, similar to categories used by Turner et al. 53 to account for the potential influence if MF exposure on tumor promotion and progression. However, the results using these alternate categorizations were unchanged (Table 3).

Duration of occupational exposure to MFs was positively associated with breast cancer in the ≥30 years exposure category in the unadjusted model, with an OR = 4.20 (95% CI = 1.52–11.56). The estimated ORs for this category remained elevated in the age‐adjusted (OR = 2.68, 95% CI = 0.96–7.55) and multivariate (OR = 2.77, 95% CI = 0.98–7.82) models.

Finally, jobs whose MF exposure could not be classified were coded as unexposed in the main analysis to maximize the available sample size for breast cancer cases. To evaluate the impact of including these jobs, a sensitivity analysis excluding jobs where MF exposure could not be classified was performed (Table 4), and results from this analysis (98 cases, 569 controls) were very similar to those presented in Table 3. Furthermore, a sensitivity analysis including socioeconomic variables (education, income, and marital status) in the multivariate model was conducted to address the potential for residual confounding by these characteristics. Results from these models were similar to the main analysis and are presented in Table S2.

**Table 4:** Sensitivity analysis of MF exposure and male breast cancer removing jobs that could not be classified for exposure

MF exposure variable	Cases, *N* (%)	Controls, *N* (%)	Age‐adjusted OR (95% CI)	Multivariate OR (95% CI)^a^
Highest average exposure				
None	73 (74)	446 (78)	1.0 (ref)	1.0 (ref)
0.3 to <0.6 *μ*T	16 (16)	94 (16)	0.96 (0.53–1.74)	0.87 (0.47–1.62)
≥0.6 *μ*T	9 (9)	29 (5)	1.98 (0.88–4.47)	1.94 (0.84–4.49)
			*P*‐trend = 0.25	*P*‐trend = 0.35
Cumulative EMF exposure^b^				
None	74 (64)	450 (79)	1.0 (ref)	1.0 (ref)
0 to <0.8	11 (10)	40 (7)	1.82 (0.87–3.81)	1.84 (0.86–3.95)
≥8.0	13 (11)	79 (14)	0.89 (0.46–1.70)	0.83 (0.42–1.63)
			*P*‐trend = 0.95	*P*‐trend = 0.91
Cumulative EMF exposure (with 5‐year lag)^c^				
None	74 (64)	454 (80)	1.0 (ref)	1.0 (ref)
0 to <0.8	10 (9)	42 (7)	1.56 (0.73–3.31)	1.57 (0.72–3.40)
≥8.0	13 (11)	73 (13)	0.92 (0.48–1.76)	0.86 (0.44–1.69)
			*P*‐trend = 0.96	*P*‐trend = 0.90
Time since last exposure				
Never exposed	73 (74)	446 (78)	1.0 (ref)	1.0 (ref)
<10 years	12 (12)	63 (11)	1.41 (0.71–2.81)	1.40 (0.69–2.82)
10–19 years	6 (6)	28 (5)	1.05 (0.41–2.70)	1.08 (0.42–2.79)
20–29 years	2 (2)	14 (2)	0.98 (0.21–4.47)	1.16 (0.25–5.36)
≥30 years	5 (5)	18 (3)	0.96 (0.34–2.72)	0.88 (0.31–2.54)
			*P*‐trend = 0.88	*P*‐trend = 0.91
Time since first exposure				
Never exposed	73 (63)	446 (78)	1.0 (ref)	1.0 (ref)
<10 years	5 (4)	20 (4)	2.81 (0.96–8.20)	2.60 (0.82–8.19)
10–19 years	4 (3)	26 (5)	1.46 (0.48–4.48)	1.41 (0.45–4.41)
20–29 years	5 (4)	40 (7)	0.76 (0.29–2.03)	0.84 (0.31–2.27)
≥30 years	11 (10)	37 (6)	1.04 (0.50–2.19)	0.89 (0.42–1.93)
			*P*‐trend = 0.93	*P*‐trend = 0.84
Age at first exposure				
Under 20	12 (12)	45 (8)	1.53 (0.76–3.08)	1.43 (0.69–2.96)
20–34	8 (8)	65 (11)	0.76 (0.34–1.66)	0.68 (0.30–1.53)
≥35	5 (5)	13 (2)	1.85 (0.63–5.45)	1.91 (0.63–5.79)
			*P*‐trend = 0.64	*P*‐trend = 0.79
Duration of exposure^d^				
Never exposed	74 (64)	479 (84)	1.0 (ref)	1.0 (ref)
<15 years	13 (11)	60 (11)	1.43 (0.73–2.77)	1.45 (0.73–2.87)
15–29 years	5 (4)	21 (4)	1.40 (0.51–3.90)	1.62 (0.57–4.64)
≥30 years	6 (5)	9 (1)	2.72 (0.92–8.01)	2.43 (0.78–7.54)
			*P*‐trend = 0.05	*P*‐trend = 0.06

MF, magnetic field; OR, odds ratio; BMI, body mass index; NECSS, National Enhanced Cancer Surveillance System.

a Adjusted for age, education, household income, marital status, BMI, and physical activity.

b Calculated as intensity of exposure × duration × full‐time status as in NECSS brain cancer analysis (Villeneuve et al. 30).

c All exposure in 5 years prior to study interview excluded.

d Part‐time and seasonal jobs weighted as half‐time of full‐time jobs.

## Discussion

Overall, this study did not detect a clear association between occupational MF exposure and breast cancer in men based on highest average exposure, although the estimated OR for a highest average MF exposure of ≥0.6 *μ*T was elevated. Our study also examined the influence of occupational MF exposure using several exposure‐time windows, specifically time since most recent (last) exposure, time since first exposure, and age at first exposure to MFs. There were no clear associations with breast cancer for any of these measures. These findings are in contrast to findings from an earlier U.S. study where an elevated risk was seen among men exposed to occupational MFs ≥30 years prior to diagnosis, particularly among men who were under age 30 at the time of this exposure 10. In addition, no associations for cumulative MF exposure and male breast cancer were observed and results were similar when a 5‐year lag was included in the cumulative exposure definition. To our knowledge, only one previous breast cancer study in men has considered a cumulative (incorporating both intensity and duration) measure of exposure to occupational MFs and did not detect an association with male breast cancer 13, similar to our results.

Using a duration of exposure metric, an increased risk of breast cancer in men was observed for employment in jobs considered above the 0.3 *μ*T average MF exposure threshold for ≥30 years. The estimated ORs for ≥30 years exposure duration in both the age and multivariate adjusted models (age‐adjusted OR = 2.68, 95% CI = 0.96–7.55; multivariate‐adjusted OR = 2.77, 95% CI = 0.98–7.82) are similar to that seen in one previous study of male breast cancer, where an OR = 2.1 (95% CI = 0.7–6.2) was observed for 30 or more years of employment in jobs exposed to MFs 10. This earlier study is the largest (most overall male breast cancer cases) of occupational MF exposure and male breast cancer that has been conducted 10 and it included similar numbers of cases and controls in the 30 or more years of exposure group as in our study. The importance of duration of exposure in the influence of MFs on cancer risk is also supported by results from a study of leukemia among Ontario Hydro workers, where duration of exposure was strongly associated with an increased risk of leukemia 54. While the similarity in results between the earlier male breast cancer study 10 and ours could suggest that long‐term occupational exposure to MFs may increase risk of breast cancer in men, the absence of a relationship for any of the other time‐related exposure metrics does not provide support for this hypothesis.

While the underlying biological mechanisms linking MF exposure with breast cancer risk has not been established 6, a role for the pineal hormone melatonin has been suggested 55, 56. Briefly, MF exposure has been hypothesized to reduce melatonin production 55, 56 and melatonin is thought to have several cancer‐protective properties 57. Most, but not all, prospective studies among women have demonstrated an inverse association between melatonin levels and breast cancer 58. To our knowledge, there have been no studies of melatonin and breast cancer in men and there is some evidence that electromagnetic field exposure has failed to produce the reductions in melatonin levels seen with night‐time light exposure in a laboratory setting 59. Furthermore, MFs have been suggested to be more relevant to tumor progression than tumor incidence, and the potential influence of duration of exposure seen in this study is consistent with a role for MF exposure in cancer progression 30.

As one of the largest studies of occupational MF exposure and breast cancer in men conducted to date 10, 11, 12, 13, 14, 15, 16, 18, we were able to examine multiple characterizations of MF exposure, a feature that has been absent from most previous studies that have used dichotomous (ever/never) measures 11, 12, 13, 14, 15, 16, 18, 20, 22, 23, 24, 27. If a long duration of exposure to MFs increases the risk of breast cancer risk in men, as suggested by our findings, this could explain why some earlier studies that did not consider exposure duration did not demonstrate associations 11, 14, 15, 16, 17, 22, 23, 24, 27. Additionally, the coding of occupational history for MF exposure in this study classified jobs according to the most likely intensity level of MF exposure (<0.3, 0.3 to <0.6, ≥0.6 *μ*T), providing a more nuanced exposure characterization than some previous studies that have only considered whether specific job types would or would not include exposure to MFs 10, 14, 16, 17, 21, 24, 27. Furthermore, individuals participating in our study provided a full occupational history for consideration and all jobs were evaluated for MF exposure. This is an improvement over earlier work where “representative” jobs from a participant's lifetime to characterize MF exposure were selected 10, 12, 17, 18, 22 such that exposure misclassification could have occurred if the selected job did not include exposure, but other jobs where an individual would have been exposed to MFs were not considered.

However, despite these strengths, this study does have some limitations. First, while the characterization of occupational MF exposure in this study was more detailed than in some previous work, no objective measures of MFs were included and characterization was based on the coding of job titles. Thus, there remains potential for exposure misclassification if individuals were employed in a job that was considered “exposed” but did not in fact have exposure to MFs over the course of their work (or vice versa). Furthermore, a much larger proportion of jobs in the male breast cancer group (18.2%) compared to the control group (0.5%) could not have their MF exposure classified. The main reason for this difference was that, due to the large number of potential controls in NECSS and the labor‐intensive nature of the occupational coding process used to characterize MF exposure, only controls with good‐quality occupational history data were included. Conversely, given that the number of male breast cancer cases was much smaller in comparison, all cases were included for job history coding, regardless of the quality of the data. This difference in quantity of missing data in the case and control groups further increases the risk of misclassification bias. However, while the main analysis included all jobs without classification as “unexposed” to maximize sample size for breast cancer cases, sensitivity analysis removing these jobs produced similar findings, suggesting that they were not driving any observed associations between MF exposure and male breast cancer. Finally, our analysis did not consider residential MF exposures, such that any individuals who had low occupational but higher residential MF exposures would be included in the unexposed group, which could have biased risk estimates toward the null. However, given that studies of breast cancer in women have not shown strong associations between residential MF exposure and breast cancer 28, 32, 33, 34, 35, it seems unlikely that the lack of consideration of potential residential exposures would greatly influence the observed results.

Additionally, while this study is one of the larger studies of breast cancer in men, there are still a relatively small number of individuals who were exposed to high levels of occupational MFs. As evidenced by the relatively wide confidence intervals associated with a number of the estimated ORs, it is possible that this study was still underpowered to detect real effects of occupational MF exposure on male breast cancer risk. Future work with larger number of cases, potentially through pooling of information across multiple male breast cancer datasets, is necessary to help clarify the relationships between occupational MF exposure and male breast cancer risk. Finally, while several established breast cancer risk factors for men were included in the multivariate model, information concerning family history of breast cancer was not available, thus the potential confounding influence of this risk factor could not be considered. However, we know of no reason to suspect that family history of breast cancer is related to occupational MF exposure, and hence it is unlikely to bias our associations.

In conclusion, while there was some suggestion that highest average exposure to MFs increases the risk of breast cancer in men, the associations with time‐related variables were less consistent and only observed with a long duration of exposure. Future research where multiple breast cancer datasets of men are pooled could be helpful to increase study power and yield more insights.

## Conflict of Interest

None declared.

## Supporting information


**Table S1.** Sensitivity analysis using alternate magnetic field index.
**Table S2.** Sensitivity analysis of magnetic field exposure and male breast cancer in multivariate models including socioeconomic status variables.
