# Supplementary material for: Occupational exposure to magnetic fields and breast cancer among Canadian men
Source: Cancer Med. 2016 Jan 21;5(3):586–96. doi: 10.1002/cam4.581 (PMC4799956; doi:10.1002/cam4.581)
Supplement: Supplementary file 1 — Table S1. Sensitivity analysis using alternate magnetic field index. Table S2. Sensitivity analysis of magnetic field exposure and male breast cancer in multivariate models including socioeconomic status variables. [file CAM4-5-586-s001.docx]

**Supplementary Table 1: Sensitivity Analysis Using Alternate Magnetic Field Index:**

| **Cumulative EMF Exposure^a^** | **Cases**  **N (%)^b^** | **Controls**  **N (%)^b^** | **Age-adjusted OR**  **(95% CI)** | **Multivariate OR**  **(95% CI)^c^** |
| --- | --- | --- | --- | --- |
| None | 75 (76%) | 450 (79%) | 1.0 (ref) | 1.0 (ref) |
| 0 - < 16.0 | 11 (11%) | 61 (11%) | 1.12 (0.55 – 2.27) | 1.09 (0.54 – 2.23) |
| ≥ 16.0 | 13 (13%) | 58 (10%) | 1.15 (0.59 – 2.22) | 1.15 (0.59 – 2.24) |
|  |  |  | p-trend = 0.64 | p-trend = 0.66 |

1. Calculated as: intensity of exposure x duration x full time status. Intensity of exposure coded as 0 for jobs with <0.3 µT, 1 for jobs with 0.3 - <0.6 µT and 4 for jobs ≥0.6 µT, similar to Koeman et al. (2013).
2. Totals may not add to n = 115 (cases) or n = 570 (controls) due to missing data
3. Adjusted for age, BMI and physical activity

**Supplementary Table 2: Sensitivity Analysis of Magnetic Field Exposure and Male Breast Cancer in Multivariate Models Including Socio-economic Status Variables:**

| **EMF Exposure Variable** | **Cases**  **N (%)** | **Controls**  **N (%)** | **Multivariate OR**  **(95% CI)^a^** |
| --- | --- | --- | --- |
| Ever EMF Exposure |  |  |  |
| None | 86 (75%) | 446 (78%) | 1.0 (ref) |
| 0.3 - <0.6 µT | 19 (17%) | 94 (17%) | 0.79 (0.43 – 1.44) |
| ≥0.6 µT | 10 (9%) | 29 (5%) | 1.81 (0.81 – 4.03) |
|  |  |  | p-trend = 0.49 |
| Cumulative EMF Exposure^b^ |  |  |  |
| None | 80 (70%) | 484 (85%) | 1.0 (ref) |
| 0 - <0.8 µT | 7 (6%) | 23 (4%) | 2.11 (0.79 – 5.59) |
| ≥ 8.0 µT | 12 (10%) | 60 (11%) | 1.03 (0.51 – 2.09) |
| Missing | 16 (14%) | 3 (0.5%) | p-trend = 0.48 |
|  |  |  |  |
| Cumulative EMF Exposure (with 5-year lag)^c^ |  |  |  |
| None | 93 (81%) | 463 (81%) | 1.0 (ref) |
| 0 - <0.8 µT | 9 (8%) | 36 (6%) | 1.40 (0.62 – 3.12) |
| ≥ 8.0 µT | 13 (11%) | 71 (13%) | 0.74 (0.38 – 1.45) |
|  |  |  | p-trend = 0.54 |
|  |  |  |  |
| Time Since Last Exposure |  |  |  |
| Never Exposed | 87 (76%) | 447 (78%) | 1.0 (ref) |
| <10 years | 13 (11%) | 63 (11%) | 1.15 (0.58 – 2.29) |
| 10 – 19 years | 7 (6%) | 28 (5%) | 0.82 (0.32 – 2.16) |
| 20 – 29 years | 3 (3%) | 14 (2%) | 1.38 (0.37 – 5.11) |
| ≥ 30 years | 5 (4%) | 18 (3%) | 0.82 (0.28 – 2.35) |
|  |  |  |  |
| Time Since First Exposure |  |  |  |
| Never Exposed | 89 (77%) | 460 (81%) | 1.0 (ref) |
| <10 years | 5 (4%) | 20 (4%) | 1.89 (0.60 – 5.95) |
| 10 – 19 years | 4 (4%) | 26 (5%) | 1.19 (0.38 – 3.66) |
| 20 – 29 years | 8 (7%) | 40 (7%) | 0.98 (0.41 – 2.33) |
| ≥30 years | 11 (10%) | 37 (7%) | 0.80 (0.37 – 1.69) |
|  |  |  | p-trend = 0.70 |
| Age at First Exposure |  |  |  |
| Under 20 | 13 (11%) | 45(8%) | 1.30 (0.65 – 2.61) |
| 20 – 29 | 7 (6%) | 50 (9%) | 0.76 (0.32 – 1.79) |
| 30 – 39 | 5 (4%) | 14 (3%) | 1.31 (0.43 – 3.98) |
| ≥ 40 | 2 (2%) | 7 (1%) | 0.47 (0.05 – 4.74) |
|  |  |  | p-trend = 0.82 |
| Duration of Exposure^d^ |  |  |  |
| Never Exposed | 89 (77%) | 480 (84%) | 1.0 (ref) |
| <15 years | 14 (12%) | 60 (11%) | 1.30 (0.67 – 2.51) |
| 15 – 29 years | 5 (4%) | 21 (3%) | 1.26 (0.45 – 3.58) |
| ≥30 years | 7 (6%) | 9 (1%) | 2.44 (0.84 – 7.15) |
|  |  |  | p-trend = 0.13 |

1. Adjusted for age, education, household income, marital status, BMI and physical activity
2. Calculated as: intensity of exposure x duration x full time status as in NECSS brain cancer analysis (Villeneuve et al., 2002)
3. All exposure in 5-years prior to study interview excluded
4. Part time and seasonal jobs weighted as half time of full time jobs
